# Cisplatin-induced oxidative stress stimulates renal Fas ligand shedding

**DOI:** 10.1080/0886022X.2018.1456938

**Published:** 2018-04-05

**Authors:** Hitesh Soni, Damian Kaminski, Rajashekhar Gangaraju, Adebowale Adebiyi

**Affiliations:** a Department of Physiology, University of Tennessee Health Science Center, Memphis, TN, USA;; b Department of Ophthalmology, University of Tennessee Health Science Center, Memphis, TN, USA;; c Department of Anatomy and Neurobiology, University of Tennessee Health Science Center, Memphis, TN, USA

**Keywords:** Renal tubules, oxidative damage, cisplatin, nephrotoxicity, captisol, Fas ligand

## Abstract

Acute kidney injury (AKI), a significant complication of cisplatin chemotherapy is associated with reactive oxygen species (ROS)-dependent renal cell death, but the cellular targets of ROS in cisplatin nephrotoxicity are not fully resolved. Here, we investigated cisplatin-induced oxidative renal damage and tested the hypothesis that ROS-dependent shedding of death activator Fas ligand (FasL) occurs in cisplatin nephropathy. We show that intraperitoneal injection of sulfobutyl ether-β-cyclodextrin (Captisol™)-solubilized cisplatin elevated the level of lipid peroxidation product malondialdehyde in mouse kidneys and urinary concentration of oxidative DNA damage biomarker 8-hydroxy-2′-deoxyguanosine. Cisplatin increased mouse kidney-to-body weight ratio and the plasma or urinary levels of predictive biomarkers of AKI, including creatinine, blood urea nitrogen, microalbumin, neutrophil gelatinase-associated lipocalin, and cystatin C. Histological analysis and dUTP nick end labeling of kidney sections indicated tubular injury and renal apoptosis, respectively in cisplatin-treated mice. Whereas the plasma concentration of soluble FasL (sFasL) was unaltered, urinary sFasL was increased ∼4-fold in cisplatin-treated mice. Real-time quantitative live-cell imaging and lactate dehydrogenase assay showed that cisplatin stimulated caspase 3/7 activation and cytotoxicity in a human proximal tubule epithelial cell line which were attenuated by inhibitors of the FasL/Fas system and poly [ADP-ribose] polymerase-1. Moreover, TEMPOL, an intracellular free radical scavenger mitigated cisplatin-induced renal oxidative stress and injury, AKI biomarker and urinary sFasL elevation, and proximal tubule cell death. Our findings indicate that cisplatin-induced oxidative stress triggers the shedding of membrane-bound FasL to sFasL in the kidney. We demonstrate that cisplatin elicits nephrotoxicity by promoting FasL/Fas-dependent oxidative renal tubular cell death.

## Introduction

Cisplatin and its platinum analogs are potent chemotherapeutic drugs used for the treatment of a wide variety of malignant tumors, including bladder, ovarian, cervical, testicular, lung, and head and neck cancers [1–3]. Cisplatin promotes cytotoxicity by forming covalent bonds with nucleophilic purine-N^7^ sites in DNA, thereby impeding DNA replication and transcription, and inducing cell death [3].

Although cisplatin is a potent chemotherapeutic agent, its use is limited by significant side effects, especially organ toxicity. Cisplatin causes neuropathy, cardiotoxicity, ototoxicity, hepatotoxicity, and nephrotoxicity [4–7]. Acute kidney injury (AKI) represents a noticeable adverse effect of cisplatin therapy as cisplatin accumulates in the kidney and causes a decline in vascular, glomerular, and tubular functions [4–7]. Given the common nephrotoxic adverse effect of cisplatin chemotherapy, elucidation of the cellular mechanisms that underlie cisplatin-induced AKI has been the focus of intense research.

Renal cell death is a central pathological mechanism of cisplatin nephrotoxicity. Following cellular uptake, cisplatin triggers the generation of a wide variety of cytotoxic mediators that promote renal tubular cell death via caspase-dependent and -independent pathways [7,8]. Fas ligand (FasL)/Fas-mediated apoptosis is one of the major cell death pathways involved in cisplatin-induced kidney injury [9–14]. Death receptor Fas and its ligand (FasL) transduce apoptotic signals via death-inducing signaling complex that activates the effector caspase-3 and -7 [15,16]. FasL and Fas expression levels were increased in tubular cells treated with cisplatin [9–13]. Accordingly, cisplatin-induced kidney injury was reduced in Fas-deficient B6-lpr/lpr mice [10]. A FasL-blocking antibody has also been shown to mitigate cisplatin nephropathy [14].

Both membrane-bound FasL (mFasL) and Fas (mFas) can be processed into soluble forms (sFasL and sFas) by metalloproteinases and alternative splicing of Fas pre-mRNA, respectively [17–19]. sFasL has been reported to be inert or less potent in promoting apoptosis compared with mFasL [20,21]. sFasL can also compete with mFasL for Fas binding, thereby inhibiting FasL/Fas signaling [21–23]. However, other studies have demonstrated that sFasL causes cell death and tissue/organ injury [24–29]. Cisplatin increased sFasL expression in cancer cells and sensitized them to sFasL-induced cytotoxicity [28,29]. Correspondingly, sequential treatment of nude mice bearing tumor grafts with cisplatin and sFasL abolished tumor growth [28]. Hence, when shed to its soluble form, FasL and cisplatin may elicit synergistic cytotoxicity [28,29]. sFasL levels are elevated in lupus nephritis and septic AKI [30,31]. However, it remains unclear whether cisplatin nephrotoxicity involves an increase in sFasL production.

Generation of reactive oxygen species (ROS) has been linked to multiple pathways that trigger cisplatin-induced organ injury [7,8,32,33]. Natural and synthetic antioxidants alleviated cisplatin-induced AKI [7,8], but the cellular targets of ROS in cisplatin nephrotoxicity are not fully resolved. ROS generation contributes to cisplatin-mediated amplification of sFasL-induced cancer cell death [28]. Oxidative stress has also been implicated in cisplatin-induced increase in renal mFasL and mFas expression [11]. In this study, we investigated cisplatin-induced oxidative renal damage and tested the hypothesis that ROS-dependent renal mFasL shedding occurs in cisplatin nephropathy.

## Materials and methods

### Animals

All experimental animal procedures were reviewed and approved by the Animal Care and Use Committee of the University of Tennessee Health Science Center (UTHSC). Male mice (C57BL/6 J; 8–10 weeks old) were purchased from the Jackson Laboratories (Bar Harbor, ME).

### HK-2 cell culture

The use of HK-2 cell line was approved, and experiments were performed in accordance with the guidelines and regulations of the Institutional Biosafety Committee of the UTHSC. Immortalized human proximal tubule epithelial cell line (HK-2; CRL-2190) was purchased from the American Type Culture Collection (Manassas, VA). The cells were maintained in complete medium consisting of keratinocyte medium supplemented with bovine pituitary extract, human recombinant epidermal growth factor (Life Technologies, Grand Island, NY), 10% fetal bovine serum, and 1% penicillin/streptomycin. The cells were cultured at 37 °C and 5% CO_2_ in a humidified incubator.

### Cisplatin preparation and in vivo studies

Cisplatin was solubilized in 20% sulfobutyl ether-β-cyclodextrin (SBE-β-CD; Captisol™) and saline by sonication for ∼20 min. A group of mice housed in a disposable and ventilated micro-isolation cage was given a single intraperitoneal (IP) injection of cisplatin (15 mg/kg). Another group of mice was pretreated with TEMPOL (100 mg/kg; IP) 1 h before cisplatin administration, followed by daily injection for 4 days. Mice in the control group were treated with Captisol. On the fifth day, urine specimens were collected from each mouse using a previously-described method [34]. Briefly, the individual mouse was weighed and placed on a new 96-well plate inside an empty Sartorius Biohit 5 mL pipette tip box for ∼2 h. After that, urine samples were collected from the wells and analyzed. Blood samples were collected via retro-orbital bleeding under isoflurane anesthesia. Mice were then euthanized using sodium pentobarbital (200 mg/kg; IP) followed by exsanguination. Kidneys were individually weighed and processed for analysis.

### Oxidative DNA damage, lipid peroxidation, and sFasL assays

Oxidative DNA damage was determined using the OxiSelect Oxidative DNA damage ELISA kit (Cell Biolabs, San Diego, CA). Urine concentration of oxidative DNA damage marker 8-hydroxy-2′-deoxyguanosine (8-OHdG) was determined according to the manufacturer’s instructions. Renal lipid peroxidation was evaluated using the Thiobarbituric Acid Reactive Substances (TBARS) kit (Cayman Chemical, Ann Arbor, MI). Malondialdehyde (MDA) levels in homogenized kidney samples were quantified. Plasma and urinary concentrations of FasL were quantified using the mouse sFasL PicoKine ELISA kit (Boster Biological Technology, Pleasanton, CA).

### Kidney injury analysis

Kidney function in mice was evaluated by measuring plasma or urinary concentration of creatinine, urea nitrogen, neutrophil gelatinase-associated lipocalin (NGAL), cystatin C, and albumin. Plasma and urinary creatinine concentrations were determined by mass spectrometry (isotope dilution LC–MS/MS) at the UAB/UCSD O’Brien Core Center for Acute Kidney Injury Research (The University of Alabama at Birmingham, Birmingham, AL). Blood urea nitrogen (BUN) level was measured using a colorimetric detection kit (Arbor Assays, Ann Arbor, MI). Urinary NGAL and cystatin C were quantified using the mouse NGAL, and cystatin C ELISA kits purchased from RayBiotech (Norcross, GA). Urinary albumin was determined using the Exocell Albuwell M kit (Exocell Inc., Philadelphia, PA).

### Histology and TUNEL assay

Kidneys samples were trimmed and processed for paraffin embedment. The samples were then sectioned for Periodic acid–Schiff (PAS) staining. The sections were subsequently analyzed by a semi-quantitative evaluation for severity of tubular injury. Slide preparations, staining, and histopathologic analysis were performed at the Probetex Inc. (San Antonio, TX). Ten fields per section were evaluated microscopically and scored based on the following scale: 0 = no apparent change; 1+ = focal: few focal areas distributed throughout the section; 2+ = infrequent: up to 8 focal areas distributed throughout the section; 3+ = frequent: up to 8 tubular profiles per 10× field; and 4+ = very frequent: more than 8 tubular profiles per 10× field. An average score per field was obtained for each group. dUTP nick end labeling (TUNEL) assay in 12 µm thick kidney sections was performed using the ApopTag Fluorescein *In Situ* Apoptosis Detection Kit (EMD Millipore, Billerica, MA).

### Live content microscopy of HK-2 cell death

Quantification of apoptosis in HK-2 cells was performed using CellPlayer caspase-3/7 reagent, and the IncuCyte ZOOM live content microscopy system (Essen BioScience, Ann Arbor, MI) as we have previously described [35]. Briefly, caspase-3/7 activation was monitored in cells plated in a 96-well microplate (∼1 × 10^4^/well). The number of green fluorescent caspase-3/7 active cells was automatically acquired and quantified at two-hourly intervals using the IncuCyte integrated analysis software.

### Lactate dehydrogenase (LDH) cytotoxicity assay

Cisplatin-induced cytotoxicity was quantified using the LDH colorimetric assay kit (Life Technologies). LDH release and percent cytotoxicity were determined according to the manufacturer’s instructions.

### Chemicals

Cisplatin, TEMPOL, Captisol, Fas blocking antibody, caspase inhibitor, and AG 14361 were obtained from EMD Millipore, Sigma-Aldrich (St. Louis, MO), CyDex Pharmaceuticals (Lenexa, KS), ProSpec-Tany TechnoGene Ltd (Rehovot, Israel), Biotium Inc. (Hayward, CA), and ApexBio (Houston, TX), respectively.

### Data analysis

Statistical analysis was performed using the InStat statistics software (GraphPad, Sacramento, CA). Statistical significance was determined using Student’s *t*-tests for paired or unpaired data and analysis of variance with Student–Newman–Keuls test for multiple comparisons. All data are expressed as mean ± standard error of mean (SEM). A *p* value <.05 was considered significant.

## Results

### Cisplatin promotes oxidative DNA damage and renal lipid peroxidation in mice

**Figure 1.**
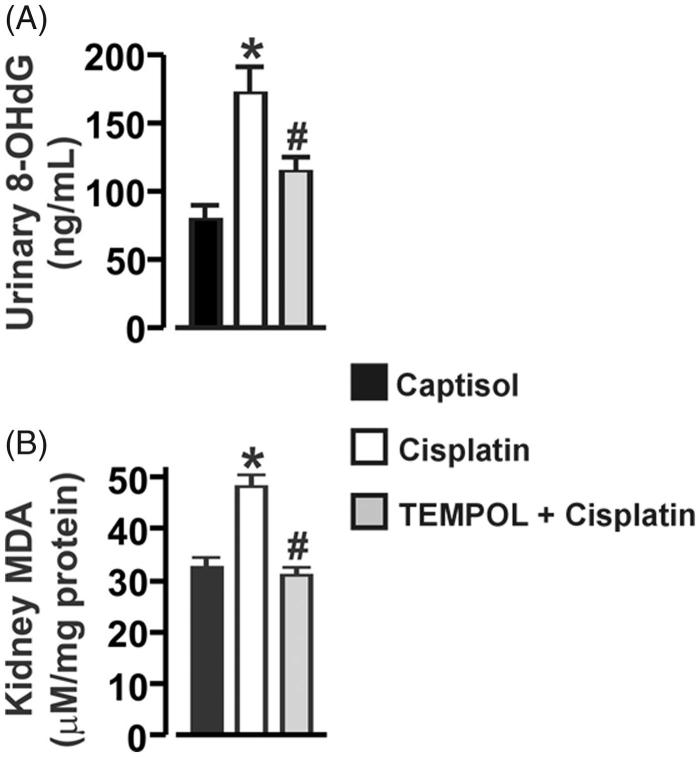
Cisplatin promotes oxidative DNA damage and renal lipid peroxidation in mice. Bar graphs summarizing: A, Urinary 8-OHdG and B, kidney MDA concentrations in Captisol (vehicle control)-, cisplatin-, and TEMPOL + cisplatin-treated mice. Mice were pretreated with TEMPOL (100 mg/kg; IP) 1 h before a single IP injection of cisplatin (15 mg/kg). Thereafter, the animals received a daily injection of TEMPOL for 4 days. *p < .05 vs. Captisol; ^#^*p* < .05 vs. cisplatin; *n* = 6 each.

To determine oxidative stress, we measured urinary and kidney concentration of 8-OHdG and MDA, respectively. As shown in Figure 1(A), the urinary 8-OHdG concentration was increased ∼2-fold in cisplatin-treated mice. Cisplatin also caused ∼1.5-fold increase in MDA level in the kidneys of the mice (Figure 1(B)). Moreover, cisplatin-induced urinary 8-OHdG and renal MDA increases were attenuated by TEMPOL, an intracellular free radical scavenger (Figure 1(A,B)). These data demonstrate that cisplatin administration causes renal oxidative stress in mice.

### Cisplatin induces ROS-dependent kidney injury in mice

**Figure 2.**
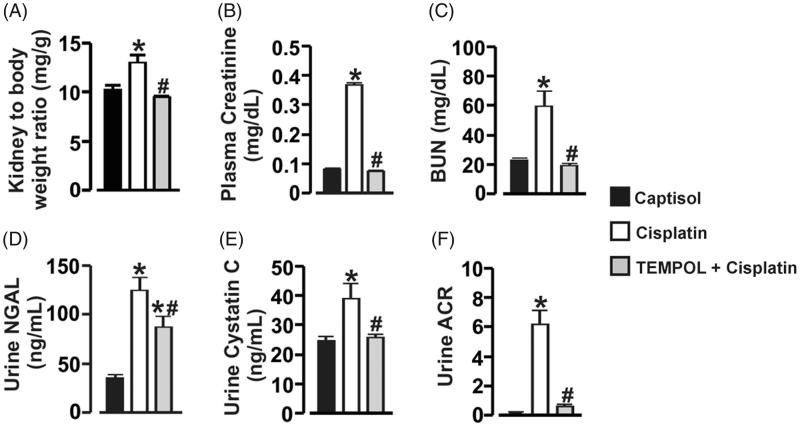
Cisplatin induces ROS-dependent kidney injury in mice. Bar graphs showing (A) kidney-to-body weight ratio, and the levels of plasma creatinine (B) and BUN (C), urinary NGAL (D), cystatin C (E), and albumin-creatinine-ratio (ACR; F) in Captisol (vehicle control)-, cisplatin (15 mg/kg; single IP injection)-, and TEMPOL (100 mg/kg; IP for 4 days) + cisplatin-treated mice. *p < .05 vs. Captisol; ^#^*p* < .05 vs. cisplatin; *n* = 6 each.

**Figure 3.**
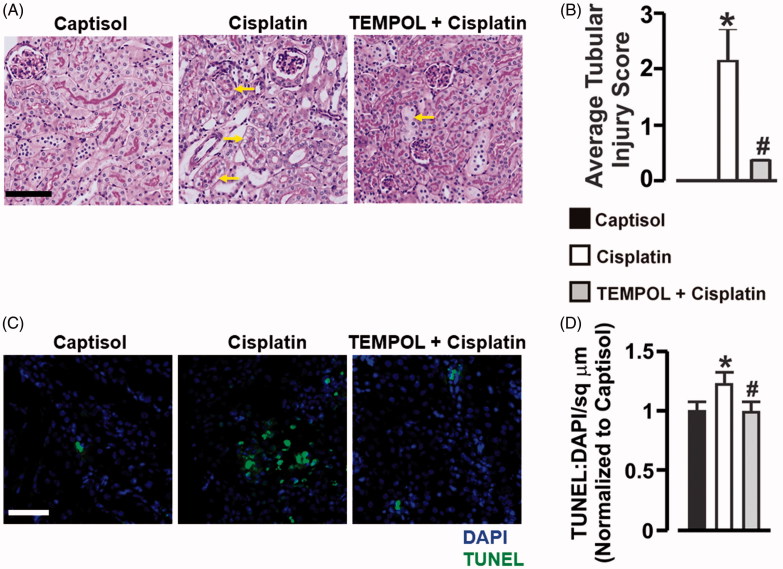
Cisplatin elicits ROS-dependent tubular damage and renal apoptosis in mice. (A) Images (PAS staining) and (B) average tubular injury score in Captisol (vehicle control)-, cisplatin (15 mg/kg; single IP injection)-, and TEMPOL (100 mg/kg; IP for 4 days) + cisplatin-treated mice. Cisplatin-treated groups showed variable tubular injury in the form of vacuolar degeneration and necrosis (arrows). However, TEMPOL ameliorated the severity of cisplatin-induced injury where few necrotic lesions were observed (arrow). (C) Representative confocal microscopy images, and (D) Bar graphs showing TUNEL staining and mean TUNEL positive cells/sq µm in Captisol (vehicle control)-, cisplatin (15 mg/kg; single IP injection)-, and TEMPOL (100 mg/kg; IP for 4 days) + cisplatin-treated mice. *p < .05 vs. Captisol; ^#^*p* < .05 vs. cisplatin; *n* = 4 each. Scale bar =50 µm.

There was no death recorded in all the groups. However, kidney-to-body weight ratio was significantly increased in cisplatin-treated mice (Figure 2(A)). To test the hypothesis that cisplatin impairs kidney function via oxidative stress in the mice, we measured the plasma or urinary levels of AKI biomarkers. Plasma creatinine and BUN concentrations were elevated ∼5-fold and 3-fold, respectively in cisplatin-treated mice (Figure 2(B,C)). Similarly, urinary NGAL, cystatin C, and albumin-creatinine-ratio (ACR) were increased ∼4-fold, 2-fold, and 38-fold, respectively (Figure 2(D–F)). TEMPOL attenuated cisplatin-induced increase in kidney-to-body weight ratio, plasma creatinine and BUN, and urinary NGAL, cystatin C, and ACR (Figure 2(A–F)). There were no apparent histological changes in the control group (Figure 3(A)). Cisplatin caused tubular injury in the form of vacuolar degeneration and necrosis (Figure 3(A,B)). However, TEMPOL ameliorated cisplatin-induced tubular damage in the mice (Figure 3(A,B)). Furthermore, TUNEL assay indicated that unlike vehicle-treated mice, apoptotic cells were extensively present in kidney sections of mice administered with cisplatin (Figure 3(C,D)). On the other hand, TEMPOL prevented cisplatin-induced renal apoptosis in the mice (Figure 3(C,D)). These findings indicate that cisplatin administration causes ROS-dependent kidney injury characterized by renal cell death.

### Cisplatin-induced oxidative stress increases urinary sFasL in mice

**Figure 4.**
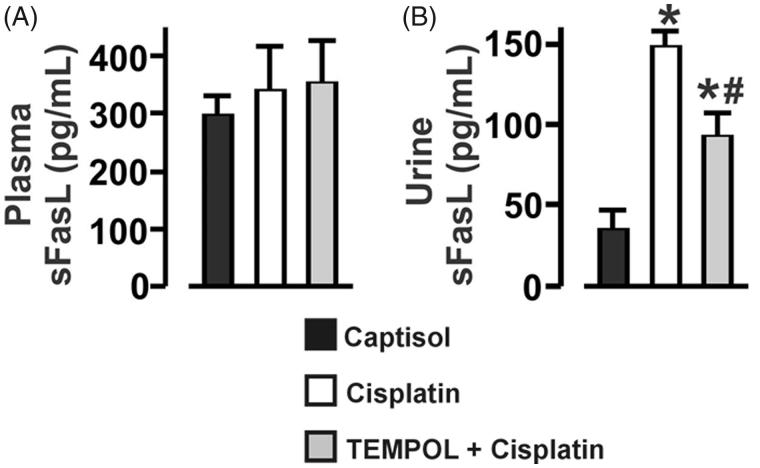
Cisplatin-induced oxidative stress increases urinary sFasL in mice. Bar graphs summarizing the levels of sFasL in: (A) Plasma, and (B) Urine of Captisol (vehicle control)-, cisplatin (15 mg/kg; single IP injection)-, and TEMPOL (100 mg/kg; IP for 4 days) + cisplatin-treated mice. *p < .05 vs. Captisol; ^#^*p* < .05 vs. cisplatin; *n* = 6 each.

The plasma concentration of sFasL was unchanged in Captisol-, cisplatin-, and TEMPOL + cisplatin-treated mice (Figure 4(A)). By contrast, urinary sFasL was increased ∼4-fold in cisplatin-treated mice. Cisplatin-induced urinary sFasL elevation was inhibited by TEMPOL (Figure 4(B)).

### ROS scavenger mitigates cisplatin-induced HK-2 cell death

**Figure 5.**
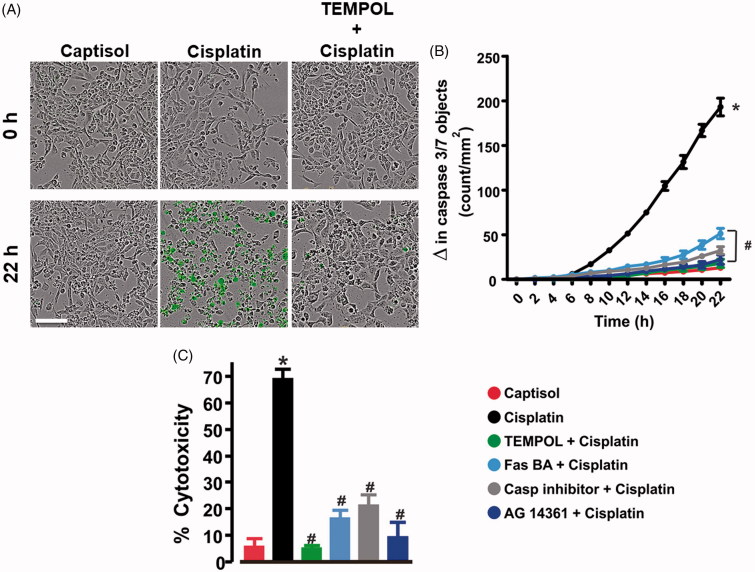
ROS scavenger mitigates cisplatin-induced human proximal tubule cell death. (A) Live content cell images (phase contrast and green fluorescent staining of nuclear DNA in apoptotic cells), and (B) kinetic curves (*n* = 5 each) demonstrating that cisplatin (30 µM) induces time-dependent increase in caspase-3/7 activity in HK-2 cells; effects abrogated by TEMPOL (1 mM), Fas blocking antibody (Fas BA; 10 µg/mL), caspase (Casp) inhibitor (Ac-DEVD-CHO; 50 µM), and AG 14361 (PARP1 inhibitor; 300 nM). (C) bar graphs (*n* = 5 each) summarizing percent cytotoxicity (LDH release) in Captisol (vehicle control)-, cisplatin (30 µM)-, and TEMPOL (1 mM) + cisplatin-, Fas BA (10 µg/mL) + cisplatin-, Casp inhibitor (50 µM) + cisplatin-, and AG 14361 (300 nM) + cisplatin-treated HK-2 cells. *p < .05 vs. Captisol; ^#^*p* < .05 vs. cisplatin. Scale bar =50 µm.

Normal healthy cells treated with the CellPlayer caspase-3/7 reagent do not exhibit fluorescence. However, apoptotic cells release the green fluorescent staining of nuclear DNA. As shown in Figure 5(A,B), cisplatin time-dependently increased caspase-3/7 activity in HK-2 cells. Significant apoptosis began 8 h after cisplatin treatment (Figure 5(B)). Pretreatment of the cells with a caspase inhibitor Ac-DEVD-CHO, Fas blocking antibody (Fas BA), poly [ADP-ribose] polymerase-1 (PARP1) inhibitor, AG 14361, and TEMPOL attenuated cisplatin-induced caspase-3/7 activation (Figure 5(A,B)). Similarly, cisplatin-induced LDH release by cultured HK-2 cells was diminished in Ac-DEVD-CHO, Fas BA, AG 14361, and TEMPOL-pretreated cells (Figure 5(C)). These data suggest that cisplatin promotes FasL-dependent renal tubular cell death via oxidative stress.

## Discussion

The salient findings of our study include 1) SBE-β-CD solubilization retains the nephrotoxic effect of cisplatin *in vivo* and *in vitro*, 2) cisplatin induces FasL-dependent renal tubular cell death via oxidative stress, and 3) cisplatin-induced oxidative stress stimulates renal sFasL production.

Platinum-based antineoplastic drugs are poorly water-soluble. Thus, cisplatin is commonly solubilized in organic solvents for use in laboratory research [36]. A recent literature review suggested that cisplatin is predominantly prepared in DMSO for research [36]. However, DMSO may interfere with the structure of platinum complexes [36,37]. Both *in vitro* and *in vivo* studies have also demonstrated that dissolution of cisplatin in DMSO inactivates its biological activities. For example, DMSO diminished the cytotoxic effects of cisplatin in cultured thyrocytes and cancer cell lines [36,38,39]. The lack of significant effect of a cisplatin formulation against appendicular osteosarcoma in dogs has also been associated with its inactivation by DMSO [40]. Therefore, solubilization of cisplatin in an appropriate medium is critical for its biological activity. To investigate cisplatin nephrotoxicity, we solubilized cisplatin in SBE-β-CD (Captisol), a pharmaceutical excipient [41–43]. Renal tubular damage and apoptosis were absent in the kidneys of Captisol-treated mice. In addition, plasma creatinine and urine ACR levels were less than 0.1 mg/dL and 0.2, respectively in the mice. These findings corroborate other studies that demonstrated an apparent lack of SBE-β-CD-induced renal toxicity [41,43].

We show that cisplatin-treated mice exhibited characteristic features of AKI, including increased levels of plasma or urinary creatinine, BUN, NGAL, cystatin C, and ACR [44,45]. Mice injected with cisplatin showed apoptotic cells with significant DNA degradation in the kidneys as evidenced by TUNEL staining. Cisplatin-induced renal apoptotic injury was paralleled by increased urinary oxidative DNA damage marker 8-OHdG and renal tubular degeneration. Cisplatin-induced renal dysfunction includes alterations in glomerular function [7]. Although light microscopy did not show histological changes in the glomeruli of mice treated with cisplatin, the presence of proteinuria suggests a failure of the glomerular filtration barrier.

Given that proximal tubules constitute a significant target of cisplatin cytotoxicity, we examined cisplatin-induced cell death in cultured HK-2 cells. We demonstrated that cisplatin promotes time-dependent activation of caspase-3/7. Furthermore, cisplatin also induced LDH release in the cells, signifying cell membrane rupture. Activation of the FasL/Fas pathway and cleavage of the nuclear protein PARP1 is known to contribute to DNA fragmentation during cell death [46,47]. Accordingly, our data show that cisplatin-induced caspase-3/7 activation and LDH release in HK-2 cells were attenuated by caspase and PARP1 inhibitors as well as Fas blocking antibody. Pharmacological inhibition or genetic ablation of FasL, Fas, and PARP1 mitigated kidney injury in cisplatin-treated mice [10,14,48,49]. Together with these previous findings, our data demonstrate the involvement of FasL/Fas-mediated extrinsic cell death signaling cascades in cisplatin nephropathy. Whereas induction of caspase-3/7 activity suggests apoptosis, LDH release indicates necrosis or cytotoxicity commonly observed during the late stage of apoptosis. Since cisplatin-induced LDH release was attenuated by caspase, PARP1, and FasL/Fas inhibitors, our data suggest the predominance of apoptosis in the cells.

TEMPOL, a membrane-permeable nitroxide metabolizes ROS by a redox cycling mechanism [50]. ROS scavenging by TEMPOL has been shown to prevent cellular and tissue injury in oxidative stress-associated cardiovascular and renal diseases [50]. The maximum tolerated dose of TEMPOL in mice when injected intraperitoneally was 275 mg/kg [51]. Here, we show that IP administration of 100 mg/kg TEMPOL to mice alleviated cisplatin-induced renal lipid peroxidation, DNA damage, AKI biomarker and urinary sFasL elevation, and proximal tubule cell death. These data are consistent with a recent study showing that orally administered TEMPOL attenuates cisplatin-induced mitochondrial-dependent oxidative stress and renal dysfunction in mice [52]. Other oxidative stress inhibitors such as sodium thiosulfate, dimethylthiourea, α-Lipoic acid, N-acetylcysteine, and vitamin C and E have been shown to be renoprotective in cisplatin-treated rodents [53–57]. Overall, these studies and our current data indicate that oxidative stress is a major mechanism that underlies cisplatin-induced nephrotoxicity.

In a previous study, cisplatin-induced increase in mFasL and mFas expression levels in rat renal epithelial cells and kidney was inhibited by a hydroxyl radical scavenger dimethylthiourea, suggesting that ROS-driven mFasL/mFas system in renal cells contribute to cisplatin-induced AKI [11]. Cisplatin treatment triggers generation of proinflammatory cytokines, including tumor necrosis factor (TNF)-alpha, which may contribute to its nephrotoxic effects [58]. FasL is a member of the TNF family, and its soluble form can mediate inflammatory reactions [59–61]. Thus, we hypothesized that cisplatin elevates the circulating level of sFasL. Surprisingly, the plasma concentration of sFasL was unaltered in cisplatin-treated mice. By contrast, the urinary level of sFasL was elevated in the mice; an effect mitigated by TEMPOL. These findings signify that 1) the circulating levels of sFasL remain the same despite cisplatin-induced renal damage, 2) urinary excretion of sFasL is associated with cisplatin-induced AKI, 3) kidney is the primary source of urinary sFasL in cisplatin-treated mice, and 4) renal oxidative stress promotes renal mFasL shedding. Perhaps the circulating level of sFasL is dependent on cisplatin dosage or treatment duration. Mechanisms by which ROS stimulate mFasL shedding are unclear. However, oxidative stress has been shown to upregulate the expression and activity of metalloproteinases [62–65]. Thus, given that metalloproteinases cleave mFasL to sFasL [17,18], ROS-driven metalloproteinase activity may contribute to renal mFasL shedding in cisplatin-treated mice. Cisplatin and sFasL can synergistically induce cytotoxicity [28]. Whether sFasL contribute to local inflammatory, pro-apoptotic, or anti-apoptotic reactions within the kidneys of cisplatin-treated mice require further investigations. Additional studies on the early phase of cisplatin nephropathy are also needed to evaluate the potential use of urinary sFasL as an early biomarker for cisplatin-induced AKI.

In conclusion, we show that cisplatin may elicit nephrotoxicity by promoting FasL-dependent oxidative renal tubular cell death. Our findings also indicate that cisplatin-induced renal oxidative stress stimulates renal mFasL shedding to sFasL. Our study supports accumulating evidence suggesting that antioxidant defense represents a potential therapeutic strategy to mitigate cisplatin nephrotoxicity.
